# Introducing ACASS: An Annotated Character Animation Stimulus Set for Controlled (e)Motion Perception Studies

**DOI:** 10.3389/frobt.2019.00094

**Published:** 2019-09-27

**Authors:** Sebastian Lammers, Gary Bente, Ralf Tepest, Mathis Jording, Daniel Roth, Kai Vogeley

**Affiliations:** ^1^Department of Psychiatry, Faculty of Medicine and University Hospital Cologne, University of Cologne, Cologne, Germany; ^2^Cognitive Neuroscience (INM-3), Institute of Neuroscience and Medicine, Research Center Jülich, Jülich, Germany; ^3^Department of Communication, Michigan State University, East Lansing, MI, United States; ^4^Human-Computer Interaction, Institute for Computer Science, University of Würzburg, Würzburg, Germany

**Keywords:** body motion, experimental paradigms, human interaction, motion capture, non-verbal behavior, social cognition, visual stimuli

## Abstract

Others' movements inform us about their current activities as well as their intentions and emotions. Research on the distinct mechanisms underlying action recognition and emotion inferences has been limited due to a lack of suitable comparative stimulus material. Problematic confounds can derive from low-level physical features (e.g., luminance), as well as from higher-level psychological features (e.g., stimulus difficulty). Here we present a standardized stimulus dataset, which allows to address both action and emotion recognition with identical stimuli. The stimulus set consists of 792 computer animations with a neutral avatar based on full body motion capture protocols. Motion capture was performed on 22 human volunteers, instructed to perform six everyday activities (mopping, sweeping, painting with a roller, painting with a brush, wiping, sanding) in three different moods (angry, happy, sad). Five-second clips of each motion protocol were rendered into AVI-files using two virtual camera perspectives for each clip. In contrast to video stimuli, the computer animations allowed to standardize the physical appearance of the avatar and to control lighting and coloring conditions, thus reducing the stimulus variation to mere movement. To control for low level optical features of the stimuli, we developed and applied a set of MATLAB routines extracting basic physical features of the stimuli, including average background-foreground proportion and frame-by-frame pixel change dynamics. This information was used to identify outliers and to homogenize the stimuli across action and emotion categories. This led to a smaller stimulus subset (*n* = 83 animations within the 792 clip database) which only contained two different actions (mopping, sweeping) and two different moods (angry, happy). To further homogenize this stimulus subset with regard to psychological criteria we conducted an online observer study (*N* = 112 participants) to assess the recognition rates for actions and moods, which led to a final sub-selection of 32 clips (eight per category) within the database. The ACASS database and its subsets provide unique opportunities for research applications in social psychology, social neuroscience, and applied clinical studies on communication disorders. All 792 AVI-files, selected subsets, MATLAB code, annotations, and motion capture data (FBX-files) are available online.

## Introduction

Observations of others' movements provide important information about our social environment. Not only do movements tell us what people are doing or what they intend to do (Dittrich, 1993; Thompson and Parasuraman, 2012; Cavallo et al., 2016), they also build the basis for far-reaching inferences about others' motivational states, moods, and emotions (Atkinson et al., 2004; Loula et al., 2005; Chouchourelou et al., 2006; Gross et al., 2012; Barliya et al., 2013). The cognitive mechanisms and the putatively distinct neural mechanisms underlying action recognition on the one hand and emotion inferences on the other hand are not yet fully understood (Vogeley, 2017). A limiting factor in previous studies has been the lack of naturalistic movement stimuli that are free of confounds and allow for high levels of experimental control (cf. Bente, 2019). This is a general requirement in motion perception studies, but particularly crucial for studies in the field of cognitive neuroscience, where distinct stimulus features that are not subject to the experimental variation, can contaminate the observed effects and aggravate their interpretation. Problematic confounds can derive from low-level physical features, such as differences in luminance or pixel changes, as well as from higher-level psychological features, such as differences in the stimulus difficulty and recognition base rates. The demand for internal validity, stands opposite to the quest for ecologically valid social stimuli, which has led to the use of more complex, real-life samples of human behavior, as captured in video documents (Bartels and Zeki, 2004; Hasson et al., 2004; Nishimoto et al., 2011; Lahnakoski et al., 2012; de Borst and de Gelder, 2015). Beyond the mentioned threats to internal validity, the disadvantage of video stimuli, in particular those collected in naturalistic settings, is evident: video documents usually disclose person variables such as age, ethnicity, gender, or attractiveness relevant to stereotypes that might interfere with inferences based on movement (Meadors and Murray, 2014). Further confounds concern the visibility of context, which has been shown to massively influence the recognition of bodily expressions (Kret and de Gelder, 2010). Last but not least, when falling back on existing media content, such as samples from TV shows or movies (Hasson et al., 2004; Spunt and Lieberman, 2012; Schmälzle et al., 2015) there is no way to control any of the visual features and no access to behavioral information of the actors, except through time consuming coding.

Different methods for stimulus production have been proposed to preserve the natural movement dynamics while avoiding the typical issues of video stimuli (cf. Bernieri et al., 1994) such as the use of point light displays (Johansson, 1973, 1976) or video quantization techniques (Berry et al., 1991, 1992). However, both methods come along with specific limitations. Although point-light displays have been shown to carry relevant information for the recognition of intentions (Manera et al., 2010) and emotions (Atkinson et al., 2004; Chouchourelou et al., 2006; Gross et al., 2012; Barliya et al., 2013; von der Lühe et al., 2016) they can only portray movements but not postural patterns (see Cutting and Proffitt, 1981), which also convey relevant emotional information (cf. Aviezer et al., 2012). Quantization techniques used to degrade video images to rougher mosaic patterns are restricted as they cannot completely obscure person characteristics, such as gender and ethnicity (see stimulus examples in Bernieri et al., 1994). These limitations can be overcome by using motion capture technologies and hereon based character animations (cf. Kret and de Gelder, 2010). Such procedures for stimulus production not only allow to systemically vary or obscure aspects of physical appearance (Bente et al., 2008, 2010) but also provide rich datasets to analyze the behavioral variations in the stimuli (Poppe et al., 2014). Importantly, we could show that character animations (lacking several visible features) produce similar impressions as videos of the original human movement they are based on (Bente et al., 2001a,b).

A setback of motion capture and character animation applications can be seen in the time consuming production process including marker application and calibration and particularly the labor intense post-production to clear the motion data from measurement artifacts and jitter before rendering. To protect these considerable investments it is reasonable to produce and publish larger stimulus data sets for multiple (re-)use. Ideally, these stimulus sets should contain annotations of low-level and high-level stimulus features, which allow other researchers to select stimulus subsets tailored to their specific research questions and methodological requirements. This is particularly true for brain imaging studies that might require the control of physical stimulus features such as brightness, contrast or pixel change dynamics in order to avoid contaminations of low-level sensory effects and high-level inferential processes. We here introduce such an annotated stimulus database suitable for the study of action recognition and emotion inferences in social perception research and social neuroscience.

**Figure 1:**
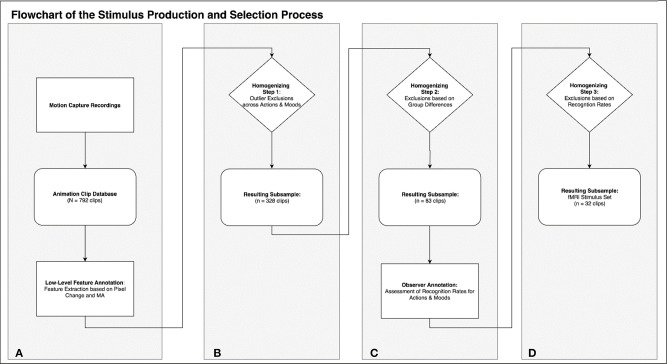
This flowchart summarizes the rationale and process of stimulus production **(A)**, annotation **(A,C)**, and selection of the stimulus subsets **(B–D)**.

Motion capture was performed on 22 human volunteers, instructed to perform six everyday activities (mopping, sweeping, painting with a roller, painting with a brush, wiping, sanding) in three different moods (angry, happy, sad; see Table 1). The six activities were chosen to be recognizable for the majority of viewers without specific expertise in contrast to movements requiring expert knowledge (e.g., particular dancing styles). Five-second clips of each motion protocol were rendered into AVI-files using two virtual camera perspectives for each clip, yielding a set of 792 stimuli. Based on this, we identified an exemplary subset of clips controlled for low- and high-level confounds: By applying a MATLAB routine for feature extraction we identified a subset of 83 clips free of outliers and characterized by maximal similarity of low-level physical stimulus features across actions and moods (see Figure 1 for an overview). In the next step we conducted an online observer study to obtain recognition rates for action and emotion which could serve as high-level psychological selection criteria for stimulus sets. Applying this data to further homogenize the stimulus set we ended with a fully balanced subset of 32 animation clips (eight variations of each of four possible combinations: two actions × two moods). This specific subset was prepared for a particular fMRI study that focused on the differential activation of the action observation network and the mentalizing system (also called theory of mind system) as related to action and emotion recognition (Geiger et al., 2019).

**Table 1:** Activities and moods recorded in the motion capture setup.

**Activities**			**Moods**
1. Mopping	2. Sweeping	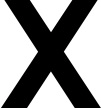	1. Happy
3. Wiping a table with a rag	4. Sanding a piece of wood on a table		2. Angry
5. Painting a wall with a brush	6. Painting a wall with a roller		3. Sad

*All six activities were performed in three designated recording blocks for each mood*.

The current article introduces the ACASS database (**A**nnotated **C**haracter **A**nimation **S**timulus **S**et) and reports the details of stimulus generation, the algorithm used for feature extraction, as well as the exemplary stepwise stimulus selection procedure leading to the subset(s). The publication includes the complete database including all animations (*N* = 792) annotated with low-level features along with two subsets: (a) with additional recognition rate annotation (*n* = 83 animations) and (b) selected for maximum homogenous and balanced properties (*n* = 32 animations). Additionally, we provide the 3D data (FBX-files, *N* = 396). Readers interested in existing motion capture databases can refer to Table 2 and the respective publications mentioned therein.

**Table 2:** Existing motion capture databases.

**Name**	**Publication**	**Availability**
The Korea University Gesture Database	Hwang, B. W., Kim, S., and Lee, S. W. (2006). A full-body gesture database for automatic gesture recognition. 7th International Conference on Automatic Face and Gesture Recognition (FGR06), 243–248. https://doi.org/10.1109/FGR.2006.8	Upon request: gesturedb@image.korea.ac.kr
The Biological Motion Library	Ma, Y., Paterson, H. M., and Pollick, F. E. (2006). A motion capture library for the study of identity, gender, and emotion perception from biological motion. Behavior Research Methods, 38(1), 134–141. https://doi.org/10.3758/BF03192758	http://paco.psy.gla.ac.uk/index.php/res/download-data
CMU Mocap Database	Not available	http://mocap.cs.cmu.edu
HDM05	Müller, M., Röder, T., Clausen, M., Eberhardt, B., Krüger, B., and Weber, A. (2007). Documentation Mocap Database HDM05 (No. CG-2007-2). Universität Bonn.	http://resources.mpi-inf.mpg.de/HDM05
HMDB	Kuehne, H., Jhuang, H., Garrote, E., Poggio, T., and Serre, T. (2011). HMDB: A large video database for human motion recognition. 2011 International Conference on Computer Vision, 2556–2563. https://doi.org/10.1109/ICCV.2011.6126543	http://serre-lab.clps.brown.edu/resource/hmdb-a-large-human-motion-database
ICS Action Database	Not available	Upon request: tmori@ics.t.u-tokyo.ac.jpOverview: http://www.miubiq.cs.titech.ac.jp/action/index.html
IEMOCAP	Busso, C., Bulut, M., Lee, C. C., Kazemzadeh, A., Mower, E., Kim, S., Narayanan, S. S. (2008). IEMOCAP: interactive emotional dyadic motion capture database. Language Resources and Evaluation, 42(4), 335. https://doi.org/10.1007/s10579-008-9076-6	Upon request: https://sail.usc.edu/iemocap/release_form.php
GEMEP Corpus	Bänziger, T., Mortillaro, M., and Scherer, K. R. (2012). Introducing the Geneva Multimodal expression corpus for experimental research on emotion perception. Emotion, 12(5), 1161–1179. https://doi.org/10.1037/a0025827	Upon request: https://www.unige.ch/cisa/gemep
The KIT whole-body human motion database	Mandery, C., Terlemez, O., Do, M., Vahrenkamp, N., and Asfour, T. (2015). The KIT whole-body human motion database. 2015 International Conference on Advanced Robotics (ICAR), 329–336. https://doi.org/10.1109/ICAR.2015.7251476	https://motion-database.humanoids.kit.edu/

*Only databases that were available to the authors are listed here. Databases that have an accompanying article but can no longer be accessed are not listed*.

## Stimulus Database

### Performers

We recruited 31 volunteers (17 females, mean age = 25.55, *SD* = 6.01) via (a) mailing lists of the study programs Psychology and Neuroscience of the University of Cologne, (b) word of mouth or (c) publicly visible notices. The volunteers which participated in the study to produce motion capture data will in the following be called “performers.” Four performers were excluded due to technical issues. Five other performers were excluded because they stated that they did not empathize sufficiently with the demanded moods during the procedure (see section Instructions and Recording-Procedures for details), resulting in a total sample of *n* = 22 (12 females, mean age = 24.73, *SD* = 4.84).

All performers were informed about the scientific background of the envisaged use of their motion capture recordings as stimulus material and gave informed consent prior to participation. All performers were either compensated monetarily (15*e*) or with credits for participation. Procedures were approved by the ethics committee of the Medical Faculty of the University of Cologne.

### Instructions and Recording-Procedures

All performers filled out a questionnaire via computer which included basic demographic information, as well as the following psychological traits (see Supplementary Data Sheet 1): a short version of the *Big Five Inventory* (Rammstedt and John, 2007), the *Toronto Empathy Questionnaire* (Spreng et al., 2009), and the *Emotional Intelligence Scale* (Schutte et al., 1998). Correlations between these traits and the subsequent recognition rates (see section Homogenizing for Recognition Rates) showed that the personality traits of the performers have no significant influence on the subsequent recognition rates when presenting the animations to naïve volunteers (Lammers, 2017).

**Figure 2:**
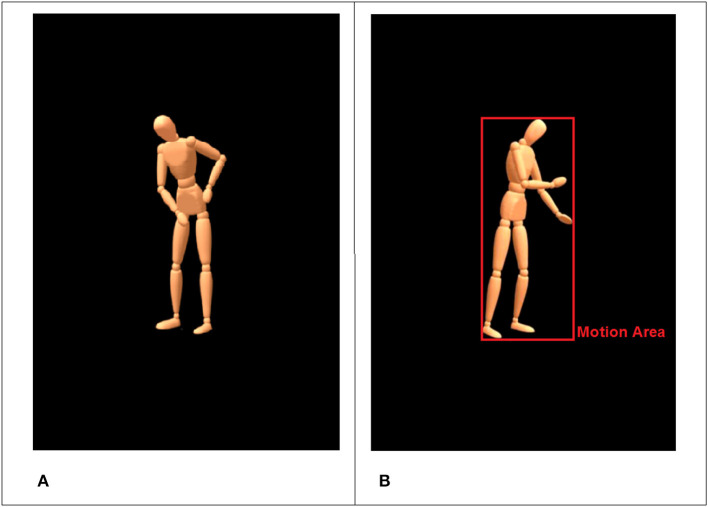
Standardized virtual character with blank face used in the animations **(A)**. The red rectangle illustrates the detected motion area for the current frame **(B)**.

We selected six everyday household activities (mopping, sweeping, painting with a roller, painting with a brush, wiping a table, sanding a piece of wood) in combination with three moods (angry, happy, sad; see Table 1) to yield animations that contain information about a specific activity (*What* *is the person doing?*) and at the same time about the underlying mood that the person was in (*How* *is the person doing it?*). The six activities can be separated in three domains (floor, table, wall) with two pairs of actions each. For instance, sweeping and mopping (floor) are not too easily differentiated when shown as wooden mannequin without the used tool (see Figure 2A).

Each volunteer performed all activities in combination with the different moods resulting in 18 recordings per performer (see Table 1). To ensure that the performers execute the different movements naturally while displaying the different moods, we used the following *mood induction procedure*. Specific instructions were presented as audio recordings to which the volunteers listened before each of the 18 recordings. Mood induction was achieved by an *Imagination Mood Induction Procedure*, which is considered to be one of the most effective ways to induce different moods (for a meta-analysis on mood induction procedures, see Westermann et al., 1996; a transcript of the instructions is provided in Supplementary Table 2).

The recordings were organized in three recording blocks according to the moods: angry, happy, and sad. The order of the three moods was randomized for each performer, while the order of activities remained the same in all three blocks. To control for immersion of the volunteers into the different moods, the performers' level of immersion into the demanded mood was assessed after each recording block via a Likert scale (*How well were you able to empathize with the required feeling?; German: Wie gut konnten Sie das von Ihnen geforderte Gefühl nachempfinden?*) ranging from 1 (*not at all*) to 11 (*very well*). The mean level of immersion was 9.197 (*SD* 1.184). Performers' data as a whole were excluded from further processing if they responded with a value equal to or smaller than five for any of the recording blocks to ensure sufficiently mood-influenced movements. Additionally, performers were asked to briefly describe the situation(s), which they imagined in order to immerse into the different moods. Directly before the next recording block they were presented with a 90 s relaxation-video (showing a tree with relaxing background music) to neutralize the mood.

### Technical Setup and Processing

The movements were recorded using an optical motion capture system with 16 infrared cameras (frame rate = 100 Hz) and the Motive Software (Optitrack™, NaturalPoint, Inc., Oregon, USA). After recordings, the 3D-data were processed and rendered using MotionBuilder® and Maya® (Autodesk Inc., California, USA) to retarget the human movements onto a virtual character in a virtual scene. We used a virtual character on a black background that looked like a wooden mannequin without a face, with detectable gross hand movements but without visibility of the fingers and the used tools (see Figure 2A).

Light sources and virtual cameras were added to all recordings in an identical fashion to ensure uniform brightness conditions. The virtual cameras defined the perspective (position, orientation, field of view) from which the resulting animation showed the mannequin. We placed two virtual cameras in each virtual scene to render the material from both the left-hand 45 degree angle and the right-hand 45 degree angle from the frontal axis. We chose this angle, because in pretests it achieved the best tradeoff between ecological validity and recognizability compared to other orientations.

From the total recording length of ~30 s only the first 5 s of the respective action were batch-rendered as PNG-files with the mental ray Plugin for Maya. We decided to use the first 5 s, because we expect the mood to be performed at peak intensity at the beginning of the recording sequence. Using a custom MATLAB script, these image-files were subsequently converted to high definition AVI-files (1280 × 720 pixels) with a frame rate of 25 frames per second.

The rendering resulted in 792 animation clips featuring 22 volunteers performing six everyday household activities in combination with three moods (see Table 1).

Additionally we provide the 396 FBX-files that allow the use in virtual reality and to further change camera angles, choose different appearances of the avatar or computations based on the 3D data.

### Low-Level Physical Feature Extraction and Stimulus Annotation

Our aim is to provide solid animation stimuli for research paradigms. As such, we deem it most important to be able to characterize the stimuli that are shown to (future) participants. While the analysis of the motion capture data would yield additional insight about the individual movements, we aimed at specifying details about the stimulus material that is presented to volunteers of future studies. This means that the analysis of the visual features of the AVI-files gains the best insight into what future participants will perceive when confronted with the stimuli.

To this end, we developed a special algorithm, which accepts most common video file formats (e.g., AVI, MPEG-1, MPEG-4). The algorithm is implemented and executed in MATLAB (R2017a, The MathWorks, Inc., Natick, USA). The routine performs a frame-by-frame comparison based on 8-bit gray-scale converted images with a black threshold of 30. The resulting signal is filtered with a moving average filter (window size = 5). The algorithm extracts two main features: (a) the size of a “*motion area*” (MA) and (b) differences in pixel intensity (i.e., pixel change). The MA is automatically defined by the 2D-area that the avatar occupies per frame and can be thought of as the smallest possible rectangle encompassing the whole body including the most distal parts (minimum bounding box). Usually these are head and feet, as well as hands, elbows or shoulders (see Figure 2B for illustration). The MA gives an impression of the extension of movements (e.g., stretched arms) and the frequency of occurring motion patterns (e.g., back and forth movements). On a more abstract level, the MA measures the size of the area in a given frame that is occupied by non-black pixels (proportion of foreground to background).

**Figure 3:**
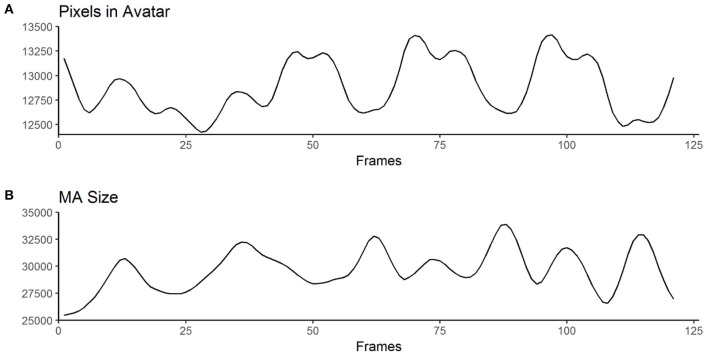
Exemplary curves computed from the raw output of the MATLAB algorithm for one animation clip. **(A)** shows an example for “Pixels in Avatar” (categories 1 – 3 in Table 2), while **(B)** displays an example for “MA-Size” (categories 4 – 6 in Table 2). The trajectories of the curves are used to derive variables such as the number of maxima or the mean amplitude (for a full list of computed variables, see Table 2 and Supplementary Table 1).

Pixel change is computed by comparing the absolute differences of gray values of each pixel frame-by-frame. This allows to infer motion parameters in general, but is particularly interesting for cases when the changes in MA are subtle (e.g., small movements in front of the body). These concepts are based on common approaches, namely *motion energy analysis* (Ramseyer and Tschacher, 2011) and *motion energy detection* (Grammer et al., 1999). The output of the *low-level feature annotation* is structured in 60 variables, with six main categories (Table 3) and 10 values each (see Supplementary Table 1). Three of the six categories are centered on pixel change computations (categories 1–3), while the other three reflect characteristics of the MA (categories 4–6). Automated curve sketching is implemented to compare the progression of motion features within and between animation clips (see Figure 3 for an example). One core element of this procedure is the translation of visible motion features into quantitative properties (e.g., number of maxima; see Supplementary Table 1, Values 3–10).

**Table 3:** Overview of Value Categories Computed by Matlab Algorithm.

**No**.	**Value category**	**Description**
1.	pixelamount	Number of non-black pixels in current frame
2.	intensitydiff	Changes of gray-scale values across time
3.	rel_intensitydiff	Amount of pixels in avatar ÷ sum of intensity differences (1 ÷ 2)
4.	MA_X	The horizontal extension of the motion area
5.	MA_Y	The vertical extension of the motion area
6.	MA_size	MA-X-Dimension × MA-Y-Dimension (4 × 5)

*To help understand the variable-names in the supplementary spreadsheets, the value categories are named accordingly here. One of the six categories always builds the first part of the variable-name. For each of these six categories, ten values (see Supplementary Table 1) were computed, resulting in a total of 60 variables. Example for the variable-name for the mean amount of pixels of a clip: pixelamount_mean. MA, motion area*.

Based on these values we defined *motion frequency* as the number of maxima of the *MA-size*-curve (e.g., how often does the avatar stretch its arms) and *motion expansiveness* as the amplitude of the *MA-size*-curve (e.g., how far does the avatar stretch its arms).

**Figure 4:**
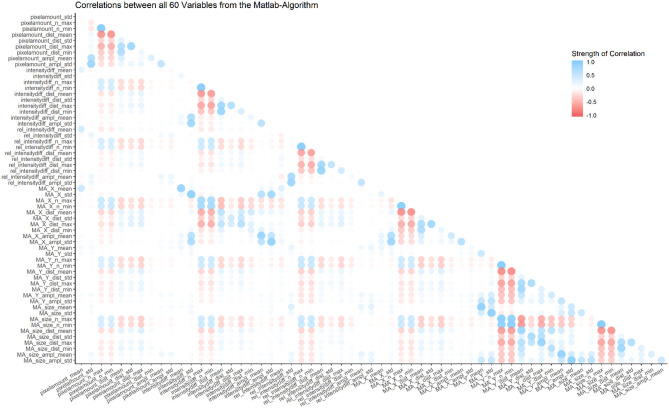
Overview of correlations between all 60 variables from the MATLAB algorithm. Most correlations are weak, but some parameters are inherently connected, and thus show strong correlations (e.g., number of minima and number of maxima in the same category).

Most of the 60 parameters show weak correlations, however some are inherently connected and thus show strong correlations (e.g., the number of maxima and the mean distance between those maxima; for a graphical representation of correlations between all parameters, see Figure 4).

### Resulting Database

**Figure 5:**
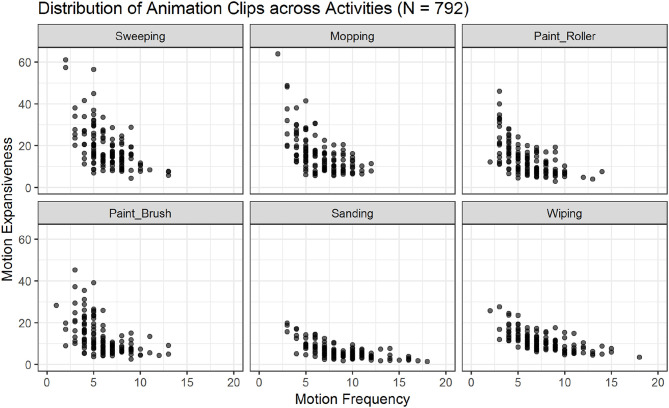
Scatter plots showing the relations of motion frequency and motion expansiveness for all six activities. Darker areas indicate the overlap of multiple animation-files. Sanding and wiping show the highest values for motion frequency, while sweeping, and mopping show the highest values for motion expansiveness.

**Figure 6:**
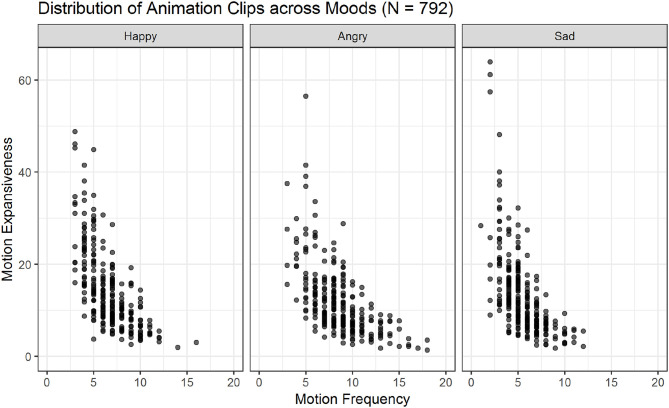
Scatter plots showing the relations of motion frequency and motion expansiveness for all three moods. Darker areas indicate the overlap of multiple animation-files. Sad movements show the highest values for motion expansiveness, while angry movements show the highest motion frequency values.

The 60 variables resulting from the low-level feature extraction were computed for all 792 animation clips and included in the database metafile (see Supplementary Data Sheet 2; see also Figures 5, 6 for an overview of all animations across actions and moods).

We used R (R Core Team, 2019), RStudio (RStudio Team, 2018) and the *lme4* package (Bates et al., 2015) to fit generalized linear mixed effects models of the relationship between motion frequency and action, as well as mood. Likelihood ratio tests were used to assess the general influence of predictors, comparing how well models including different predictors fit a given data set while taking into account the models' complexity. The significance of the effect of each predictor was tested by comparing a model including the predictor with the same model without the predictor against a significance level of 0.05.

*Post hoc* tests were computed for the comparison between factor levels (correcting for multiple comparisons) with the *glht()* function from the *multcomp* package (Hothorn et al., 2008). To analyze motion frequency, a model including action and mood (without interaction term) as fixed effects with random intercepts for motion capture performers was fitted and performed significantly better than the null model including only the intercept or models with only one of the fixed effects [χ^2^_(2)_ = 176.31, *p* < 0.001].

**Figure 7:**
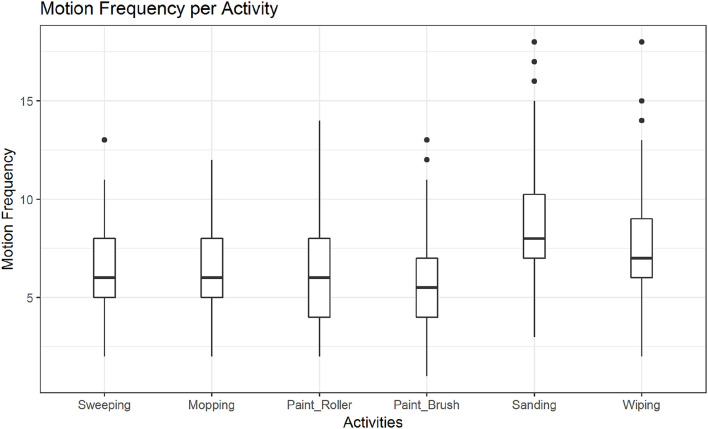
Distribution of motion frequency across activities. A significant difference is found between the mean motion frequency of sanding vs. wiping (*p* < 0.01).

**Figure 8:**
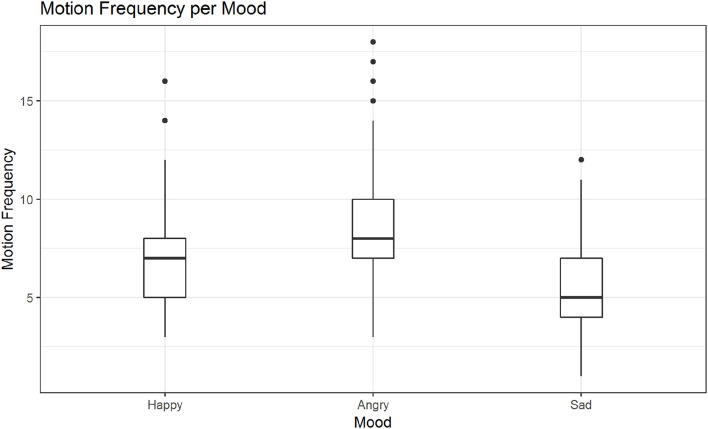
Distribution of motion frequency across moods. Significant differences are found in the mean motion frequency between happy and sad movements, angry and sad movements and notably also between happy and angry movements (in all mentioned contrasts: *ps < 0.001*).

In *post hoc* tests we found significant differences in the mean motion frequency for sanding vs. wiping (*M* = −*0.14, SE* = *0.04, z* = −*3.16, p* < 0.01; see also Figure 7), but not between the two other pairs of activities. The tests further revealed significant differences in the mean motion frequency between happy and sad movements, *M* = −*0.19, SE* = *0.04, z* = −*5.28, p* < 0.001, angry and sad movements, *M* = −*0.44, SE* = *0.03, z* = −*12.99, p* < 0.001 and notably also between happy and angry movements, *M* = *0.25, SE* = *0.03, z* = *7.84, p* < 0.001 (see also Figure 8).

## Defining Stimulus Subsets

In the following we exemplarily demonstrate a stimulus selection procedure which results in an optimal set to compare neural correlates of action and emotion recognition. This selection is based on the low level video features described above, as well as on an additional annotation based on observer recognition rates for actions and emotions (see section Homogenizing for Recognition Rates). The procedure comprises three selection steps, which lead to a highly homogenous set of 32 stimuli with eight clips for each of the four different possible combinations (two actions × two emotions; see Figures 1B–D for an overview of the selection procedure).

### Homogenizing for Low-Level Physical Features

#### Procedure

**Figure 9:**
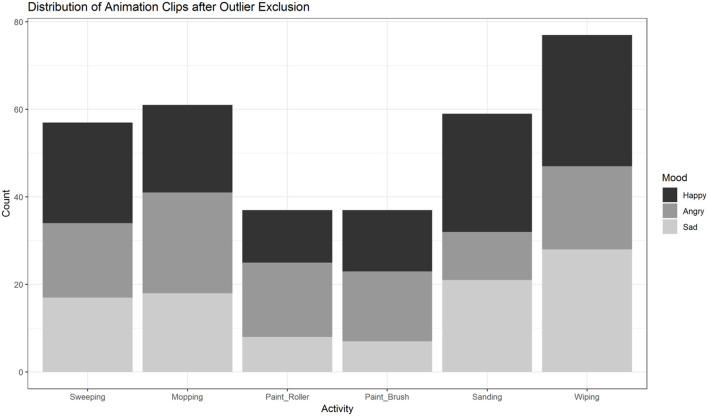
Distribution of animation clips (*n* = 328) across activities and moods after exclusion of outliers based on low-level feature extraction. Painting-activities were excluded significantly more often than the four other activities. The distribution of sanding and wiping across moods is unbalanced compared to sweeping and mopping.

First, we excluded single animation clips with outliers in any of the 60 variables (outlier defined as a value outside the range of *M*±2 × *SD*) to ensure comparability across action and emotion categories. To this end a z-score for each variable was computed. After excluding clips with outlier data in any of the 60 variables, 328 of the initial 792 animations remained (see Figure 1B). The distribution of the remaining clips across conditions (actions, moods) is illustrated in Figure 9. In a second step, the remaining 328 videos were subsequently analyzed with R (R Core Team, 2019) and RStudio (RStudio Team, 2018) in (generalized) linear mixed effects models, followed by *post hoc* tests as described above.

The goal was to remove groups that show significant differences in their motion frequency and to identify the subset of clips with the highest possible homogeneity (see Figure 1C). Since motion frequency is reported to be the most characteristic parameter of movements under varying emotional conditions (Paterson et al., 2001; Sawada et al., 2003), we decided to focus on this variable in the selection process. The results for motion expansiveness are reported as an additional descriptive parameter.

## Results

Motion frequency was analyzed in generalized linear mixed effects models with action and mood as fixed effects and random intercepts for motion capture performers. A model including action and mood (without interaction term) as fixed effects fitted the data significantly better than the null model including only the intercept or models with only one of the fixed effects [χ^2^_(2)_ = 16.67, *p* < 0.001].

Even after filtering outliers there were still significant differences between sad and happy activities, *M* = −*0.13, SE* = *0.05, z* = −*2.52, p* < *0.05*, as well as sad and angry actions, *M* = −*0.22, SE* = *0.05, z* = −*4.05, p* < *0.001*. No significant difference was found between happy and angry actions, *M* = *0.09, SE* = *0.05, z* = *1.82, p* = *0.16*. Hence animations containing sad actions were excluded, to homogenize the stimulus set with respect to motion frequency.

In contrast to the analysis prior to the exclusion of outliers, the *post hoc* tests now did not show any significant differences between the motion frequency of either of the three pairs of activities (floor, table, wall). Painting activities were excluded more often by the procedure of outlier removals (see Figure 9). In the four other actions (domains: floor, table) there was an uneven distribution among sanding and wiping across moods (see Figure 9). Thus, we decided to exclude table- and wall-activities.

Motion expansiveness was investigated by comparing the fit of linear mixed effects models with random intercepts for motion capture performers. A model including action as fixed effect fitted the data significantly better than the null model including only the intercept [χ^2^_(5)_ = 123.90, *p* < 0.001]. Adding mood as fixed effect (without interaction term) did not significantly improve the model fit [χ^2^_(2)_ = 1.94, *p* = 0.38] and was thus not included in the model.

*Post hoc* tests revealed significant differences between mopping and sweeping, *M* = −*3.38, SE* = *0.84, z* = −*4.04, p* < *0.001*, as well as between sanding and wiping, *M* = *4.23, SE* = *0.78, z* = *5.40, p* < *0.001*, but no significant difference between the two painting-activities, *M* = −*0.19, SE* = *1.05, z* = −*0.18, p* = *0.99*.

On the basis of these arguments we decided to focus the following steps on a 2 × 2 design with the actions being mopping vs. sweeping, and the moods being happy vs. angry (*n* = 83 remaining clips).

### Homogenizing for Recognition Rates

This particular selection was intended for a functional neuroimaging study where task difficulty across conditions was ideally balanced between both tasks (Geiger et al., 2019). We therefore conducted an online survey using the remaining 83 clips to receive an additional annotation for these animations. In this survey we showed each animation to volunteers to compute recognition rates for actions and moods. Taking recognition rates as estimate of task difficulty, we further selected clips to homogenize for this high-level feature (see Figure 1D). This is especially important in cognitive neuroscience studies to avoid confounding effects of task difficulty on observed brain activity.

#### Participants (Observers)

We recruited 112 volunteers (73 females, mean age = 31.66, *SD* = 11.71) independently from the group of performers (see section Performers) via (a) mailing lists of the study programs Biology, Neuroscience, Philosophy and Psychology of the University of Cologne, (b) word of mouth or (c) a designated mailing list of volunteers of the Research Center Jülich.

Three participants who's answering behavior differed significantly (deviations >2 × *SD*) from the rest of volunteers were excluded. Additionally, six participants were excluded because they were presented with too many incomplete animations (>2 × *SD*). The number of incomplete animation playbacks was dependent on the computer hardware and internet connection of each participant. To ensure that the majority of ratings are based on the viewing of complete animations, we excluded participants' ratings with many incomplete animation playbacks. Four participants were excluded, because of technical difficulties, resulting in a total remaining sample of *n* = 99 (64 females, mean age = 31.52, *SD* = 12.03).

#### Procedure

At the beginning of the survey, all participants received structured instructions. It was pointed out that all data were collected and analyzed anonymously. It was further emphasized that the task was either to focus on (a) the action or (b) the mood displayed. Tasks were always indicated before the start of the video and were additionally displayed above the video during its presentation. After the presentation, participants were prompted with an explicit forced-choice format [for the activity: (a) mopping or (b) sweeping; for the mood: (a) happy or (b) angry]. The animations were divided into four subgroups, containing either 20 or 21 clips with approximately equal amounts of clips per mood and activity. Each volunteer was randomly assigned to one of four subgroups and rated each animation of that subgroup for activity and mood. The order of the clips was randomized within the subgroups. After completing the video ratings, basic information (age, gender, handedness, sportiness, years of education) was assessed. The experiment was finished with a short debriefing that informed the participants about the general purpose of the survey and the overarching project. The recognition rates were computed by dividing the amount of correct answers by the total amount of given answers for each animation (for both activities and moods). The survey was conducted via Unipark (Questback GmbH, EFS Survey, Version 10.9, http://www.unipark.com). Results were analyzed in SPSS (Version 24). For the purpose of data cleansing, z-scores were computed for (a) responses, (b) the amount of incomplete clips (see section Participants (Observers) for details).

#### Results

**Figure 10:**
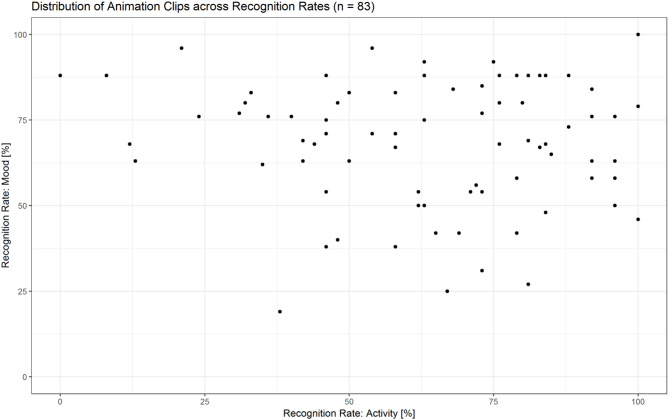
Distribution of animation clips (*n* = 83) across recognition rates for activities and moods. The majority of animations were recognized above chance level within a range from 55–100% correctness in at least one condition.

The majority of animations were rated above chance level within a range from 55 to 100% correctness in at least one condition (see Figure 10 and Supplementary Data Sheet 3). Thirty-six clips were rated both for action as well as mood at a rate of ≥55%, with a maximum accuracy difference of 30 percentage points between the two scores. For the selection of the fMRI stimulus set, we controlled for two parameters: (a) difference between the two recognition rates (<30 percentage points), (b) equal amount of combinations between activities and moods [angry mopping (*n* = 8), angry sweeping (*n* = 8), happy mopping (*n* = 8), happy sweeping (*n* = 8); see Supplementary Data Sheet 4].

## Discussion and Future Prospects

We herewith present the ACASS database including 792 animations with their respective annotations about basic motion features and emotional expressions inscribed therein. The outstanding features of this newly generated database are (a) the uniform presentation across actors after transferring all human movements onto the same avatar and (b) the motion feature annotation of all animations. The low-level physical feature annotation allows to define various subsets, for instance selecting maximum heterogeneous or homogenous subsets. Furthermore, additional annotations, for instance regarding psychological evaluations as provided by neutral observers can enrich the database and extend its usefulness even beyond the possible applications sketched here.

As a show case, we have demonstrated here as one example how to extract a homogeneous stimulus subset with respect to perceived difficulty of action and mood recognition for the purpose of a particular functional neuroimaging study in the field of social cognitive neuroscience that aimed at identifying the neural correlates of action recognition and mood recognition (Geiger et al., 2019).

For this subset of the database, different types of application within social neuroscience come to mind: it would be very interesting and timely to investigate the temporal relations of the involved brain systems with more suitable technology like magnetoencephalography. Another obvious question is that of functional connectivity of the involved brain regions. This leads to questions about changes in psychopathological conditions. Abnormalities have been reported for mentalizing abilities in conditions such as schizophrenia and autism spectrum disorders (Frith, 2004). Functional connectivity has been shown to be altered between and within the mentalizing system and the action observation network in autism spectrum disorders (Fishman et al., 2014). With our novel stimulus subset the neural correlates of the involved systems can be investigated in more detail.

Aside from possible applications in the field of social cognitive neuroscience, the stimulus subset, as well as other individually chosen subsets from the database can serve in behavioral studies that use the annotational information to systematically vary e.g., task difficulty (recognition rates). For instance, this could be interesting to contrast ambiguous animations with recognition rates close to guessing rate with other animations that are mostly correctly recognized according to the observer annotation. A further interesting study could be to examine animations that are easily recognized for only one category (e.g., action but not mood). A free viewing task could be conducted to see what the spontaneous attributions of observers are, when no specific instructions and answering options are given. The stimuli could be further enhanced to use in studies about perspective taking and embodiment, e.g., by use in virtual reality or systematically varying the camera angle. Another interesting line of investigation could be to ask participants to rate animations for valence and arousal.

The ACASS database, including the subsets, as well as the source code of the algorithm are hosted at FigShare (doi.org/10.6084/m9.figshare.c.4443014) (preview during review-process). Annotational information are provided in designated CSV-files to enable the selection of individual sets of animations.

## Limitations

The ACASS database contains recordings of six different household activities that we expect the vast majority of viewers to recognize. All activities were performed stand-alone. Thus, the recordings do not cover interactive situations like dyadic activities or those that address the viewer as an interaction partner. Our main field of application is aimed to be person perception as a well-established domain in social psychology, which includes the processing of social information derived from mere observation beyond true interactions.

## Data Availability Statement

All datasets generated for this study are included in the manuscript and the Supplementary Files.

## Ethics Statement

This study was carried out in accordance with the recommendations of the ethics committee of the Medical Faculty of the University of Cologne with written informed consent from all subjects. All subjects gave written informed consent in accordance with the Declaration of Helsinki. The protocol was approved by the ethics committee of the Medical Faculty of the University of Cologne.

## Author Contributions

GB and KV conceived the project. DR and SL prepared the motion capture recordings. SL conducted the motion capture recordings and prepared the first draft of the manuscript. RT developed the MATLAB algorithm. SL and MJ analyzed the data. All authors reviewed and edited the manuscript.

### Conflict of Interest

The authors declare that the research was conducted in the absence of any commercial or financial relationships that could be construed as a potential conflict of interest.
