# Supplementary material for: Introducing ACASS: An Annotated Character Animation Stimulus Set for Controlled (e)Motion Perception Studies
Source: Front Robot AI. 2019 Sep 27;6:94. doi: 10.3389/frobt.2019.00094 (PMC7805965; doi:10.3389/frobt.2019.00094)
Supplement: Supplementary file 5 [file Table_1.DOCX]

Supplementary Material

# Supplementary Table 1: Overview of Values Computed by Matlab Algorithm for Each Value Category

*Note.* To help understand the variable-names in the spreadsheets, the values are named accordingly here. One of the ten values always builds the second part of the variable-name. These ten values were computed for each of the six value categories (see Table 2) resulting in a total of 60 variables. Example for the variable-name of the mean MA-Size of a clip: *MA_size_mean*. MA = motion area.

| No. | Value | Description |
| --- | --- | --- |
| I. | mean | Mean of value category (e.g. MA-Size) |
| II. | std | Standard deviation of value category |
| III. | n_max | Number of maxima (for whole length of video) |
| IV. | n_min | Number of minima (for whole length of video) |
| V. | dist_mean | Mean distance between maxima |
| VI. | dist_std | Standard deviation of the distance between maxima |
| VII. | dist_max | Maximum distance between maxima |
| VIII. | dist_min | Minimum distance between maxima |
| IX. | ampl_mean | Mean amplitude (i.e. mean height difference of maxima & minima) |
| X. | ampl_std | Standard deviation of the amplitude |
