# Supplementary material for: Introducing ACASS: An Annotated Character Animation Stimulus Set for Controlled (e)Motion Perception Studies
Source: Front Robot AI. 2019 Sep 27;6:94. doi: 10.3389/frobt.2019.00094 (PMC7805965; doi:10.3389/frobt.2019.00094)
Supplement: Supplementary file 6 [file Table_2.DOCX]

Supplementary Material

# Transcript 1: Transcripts of the Audio-Instructions

This is a transcript of the audio instructions used during the motion capture recordings to induce the three different moods and specify the respective activity. There is a specific instruction for each of the 18 combinations of activity and mood. All instructions are first presented in the original version (german) and a subsequent english translation.

Angry sweeping:

German: *Stell dir möglichst detailliert vor, dir passiert etwas, das dich richtig wütend macht. So richtig, richtig wütend. Versuche dich so gut wie möglich in dieses Gefühl hineinzuversetzen. Sobald du dich in diese Lage versetzt hast, bitten wir dich die Fläche vor dir zu fegen.*

English: *Imagine as detailed as possible, something happens to you that makes you really angry. Really, really angry. Try to put yourself as good as possible into this feeling. As soon as you have put yourself in this position, we ask you to sweep the area in front of you.*

Angry mopping:

German: *Stell dir erneut detailliert vor, dir passiert etwas, das dich richtig wütend macht. So richtig, richtig wütend. Versuche dich so gut wie möglich in dieses Gefühl hineinzuversetzen. Sobald du dich in diese Lage versetzt hast, bitten wir dich die Fläche vor dir zu wischen.*

English: *Imagine again in detail something happening to you that makes you really angry. Really, really angry. Try to put yourself as good as possible into this feeling. As soon as you have put yourself in this position, we ask you to mop the area in front of you.*

Angry painting (brush):

German: *Stell dir erneut detailliert vor, dir passiert etwas, das dich richtig wütend macht. So richtig, richtig wütend. Versuche dich so gut wie möglich in dieses Gefühl hineinzuversetzen. Sobald du dich in diese Lage versetzt hast, bitten wir dich die Wand mit dem Pinsel anzumalen.*

English: *Imagine again in detail something happening to you that makes you really angry. Really, really angry. Try to put yourself as good as possible into this feeling. As soon as you have put yourself in this position, we ask you to paint the wall with the brush.*

Angry painting (roller):

German: *Stell dir erneut detailliert vor, dir passiert etwas, das dich richtig wütend macht. So richtig, richtig wütend. Versuche dich so gut wie möglich in dieses Gefühl hineinzuversetzen. Sobald du dich in diese Lage versetzt hast, bitten wir dich die Wand mit der Rolle anzumalen.*

English: *Imagine again in detail something happening to you that makes you really angry. Really, really angry. Try to put yourself as good as possible into this feeling. As soon as you have put yourself in this position, we ask you to paint the wall with the roller.*

Angry sanding:

German: *Stell dir erneut detailliert vor, dir passiert etwas, das dich richtig wütend macht. So richtig, richtig wütend. Versuche dich so gut wie möglich in dieses Gefühl hineinzuversetzen. Sobald du dich in diese Lage versetzt hast, bitten wir dich den Holzblock abzuschleifen.*

English: *Imagine again in detail something happening to you that makes you really angry. Really, really angry. Try to put yourself as good as possible into this feeling. As soon as you have put yourself in this position, we ask you to sand the wooden block.*

Angry wiping:

German: *Stell dir erneut detailliert vor, dir passiert etwas, das dich richtig wütend macht. So richtig, richtig wütend. Versuche dich so gut wie möglich in dieses Gefühl hineinzuversetzen. Sobald du dich in diese Lage versetzt hast, bitten wir dich den Tisch mit dem Lappen zu wischen.*

English: *Imagine again in detail something happening to you that makes you really angry. Really, really angry. Try to put yourself as good as possible into this feeling. As soon as you have put yourself in this position, we ask you to wipe the table with the rag.*

Happy sweeping:

German: *Stell dir möglichst detailliert vor, dir passiert etwas, das dich richtig glücklich macht. So richtig, richtig glücklich. Versuche dich so gut wie möglich in dieses Gefühl hineinzuversetzen. Sobald du dich in diese Lage versetzt hast, bitten wir dich die Fläche vor dir zu fegen.*

English: *Imagine as detailed as possible, something happens to you that makes you really happy. Really, really happy. Try to put yourself as good as possible into this feeling. As soon as you have put yourself in this position, we ask you to sweep the area in front of you.*

Happy mopping:

German: *Stell dir erneut detailliert vor, dir passiert etwas, das dich richtig glücklich macht. So richtig, richtig glücklich. Versuche dich so gut wie möglich in dieses Gefühl hineinzuversetzen. Sobald du dich in diese Lage versetzt hast, bitten wir dich die Fläche vor dir zu wischen.*

English: *Imagine again in detail something happening to you that makes you really happy. Really, really happy. Try to put yourself as good as possible into this feeling. As soon as you have put yourself in this position, we ask you to mop the area in front of you.*

Happy painting (brush):

German: *Stell dir erneut detailliert vor, dir passiert etwas, das dich richtig glücklich macht. So richtig, richtig glücklich. Versuche dich so gut wie möglich in dieses Gefühl hineinzuversetzen. Sobald du dich in diese Lage versetzt hast, bitten wir dich die Wand mit dem Pinsel anzumalen.*

English: *Imagine again in detail something happening to you that makes you really happy. Really, really happy. Try to put yourself as good as possible into this feeling. As soon as you have put yourself in this position, we ask you to paint the wall with the brush.*

Happy painting (roller):

German: *Stell dir erneut detailliert vor, dir passiert etwas, das dich richtig glücklich macht. So richtig, richtig glücklich. Versuche dich so gut wie möglich in dieses Gefühl hineinzuversetzen. Sobald du dich in diese Lage versetzt hast, bitten wir dich die Wand mit der Rolle anzumalen.*

English: *Imagine again in detail something happening to you that makes you really happy. Really, really happy. Try to put yourself as good as possible into this feeling. As soon as you have put yourself in this position, we ask you to paint the wall with the roller.*

Happy sanding:

German: *Stell dir erneut detailliert vor, dir passiert etwas, das dich richtig glücklich macht. So richtig, richtig glücklich. Versuche dich so gut wie möglich in dieses Gefühl hineinzuversetzen. Sobald du dich in diese Lage versetzt hast, bitten wir dich den Holzblock abzuschleifen.*

English: *Imagine again in detail something happening to you that makes you really happy. Really, really happy. Try to put yourself as good as possible into this feeling. As soon as you have put yourself in this position, we ask you to sand the wooden block.*

Happy wiping:

German: *Stell dir erneut detailliert vor, dir passiert etwas, das dich richtig glücklich macht. So richtig, richtig glücklich. Versuche dich so gut wie möglich in dieses Gefühl hineinzuversetzen. Sobald du dich in diese Lage versetzt hast, bitten wir dich den Tisch mit dem Lappen zu wischen.*

English: *Imagine again in detail something happening to you that makes you really happy. Really, really happy. Try to put yourself as good as possible into this feeling. As soon as you have put yourself in this position, we ask you to wipe the table with the rag.*

Sad sweeping:

German: *Stell dir möglichst detailliert vor, dir passiert etwas, das dich richtig traurig macht. So richtig, richtig traurig. Versuche dich so gut wie möglich in dieses Gefühl hineinzuversetzen. Sobald du dich in diese Lage versetzt hast, bitten wir dich die Fläche vor dir zu fegen.*

English: *Imagine as detailed as possible, something happens to you that makes you really sad. Really, really sad. Try to put yourself as good as possible into this feeling. As soon as you have put yourself in this position, we ask you to sweep the area in front of you.*

Sad mopping:

German: *Stell dir erneut detailliert vor, dir passiert etwas, das dich richtig traurig macht. So richtig, richtig traurig. Versuche dich so gut wie möglich in dieses Gefühl hineinzuversetzen. Sobald du dich in diese Lage versetzt hast, bitten wir dich die Fläche vor dir zu wischen.*

English: *Imagine again in detail something happening to you that makes you really sad. Really, really sad. Try to put yourself as good as possible into this feeling. As soon as you have put yourself in this position, we ask you to mop the area in front of you.*

Sad painting (brush):

German: *Stell dir erneut detailliert vor, dir passiert etwas, das dich richtig traurig macht. So richtig, richtig traurig. Versuche dich so gut wie möglich in dieses Gefühl hineinzuversetzen. Sobald du dich in diese Lage versetzt hast, bitten wir dich die Wand mit dem Pinsel anzumalen.*

English: *Imagine again in detail something happening to you that makes you really sad. Really, really sad. Try to put yourself as good as possible into this feeling. As soon as you have put yourself in this position, we ask you to paint the wall with the brush.*

Sad painting (roller):

German: *Stell dir erneut detailliert vor, dir passiert etwas, das dich richtig traurig macht. So richtig, richtig traurig. Versuche dich so gut wie möglich in dieses Gefühl hineinzuversetzen. Sobald du dich in diese Lage versetzt hast, bitten wir dich die Wand mit der Rolle anzumalen.*

English: *Imagine again in detail something happening to you that makes you really sad. Really, really sad. Try to put yourself as good as possible into this feeling. As soon as you have put yourself in this position, we ask you to paint the wall with the roller.*

Sad sanding:

German: *Stell dir erneut detailliert vor, dir passiert etwas, das dich richtig traurig macht. So richtig, richtig traurig. Versuche dich so gut wie möglich in dieses Gefühl hineinzuversetzen. Sobald du dich in diese Lage versetzt hast, bitten wir dich den Holzblock abzuschleifen.*

English: *Imagine again in detail something happening to you that makes you really sad. Really, really sad. Try to put yourself as good as possible into this feeling. As soon as you have put yourself in this position, we ask you to sand the wooden block.*

Sad wiping:

German: *Stell dir erneut detailliert vor, dir passiert etwas, das dich richtig traurig macht. So richtig, richtig traurig. Versuche dich so gut wie möglich in dieses Gefühl hineinzuversetzen. Sobald du dich in diese Lage versetzt hast, bitten wir dich den Tisch mit dem Lappen zu wischen.*

English: *Imagine again in detail something happening to you that makes you really sad. Really, really sad. Try to put yourself as good as possible into this feeling. As soon as you have put yourself in this position, we ask you to wipe the table with the rag.*
